# Association of Genetic Variation in the Epithelial Sodium Channel Gene with Urinary Sodium Excretion and Blood Pressure

**DOI:** 10.3390/nu10050612

**Published:** 2018-05-14

**Authors:** Yoon Jung Yang, Jihye Kim, Chang Keun Kwock

**Affiliations:** 1 Department of Foods and Nutrition, College of Natural Sciences, Dongduk Women’s University, Seoul 02748, Korea; yjyang@dongduk.ac.kr; 2 Department of Preventive Medicine, College of Medicine, Hanyang University, Seoul 04764, Korea; eluzai81@hanyang.ac.kr; 3 Nutrition and Diet Research Group, Korea Food Research Institute, Jeollabuk-do 55365, Korea

**Keywords:** ENaC, genetic variant, *SCNN1A*, *SCNN1B*, *SCNN1G*, blood pressure, 24-h urinary sodium excretion

## Abstract

This study was performed to investigate whether genetic variation in the epithelial sodium channel (ENaC) is associated with 24-h urinary sodium excretion and blood pressure. A total of 3345 participants of the KoGES_Ansan and Ansung study were eligible for this study. Genomic DNA samples were isolated from peripheral blood and genotyped on the Affymetrix Genome-Wide Human SNP Array 5.0. Thirty-four single nucleotide polymorphisms (SNPs) were extracted for gene regions (*SCNN1A*, *SCNN1B*, and *SCNN1G*) as additive components by using Plink. Twenty-four-hour sodium excretions were estimated from spot urine samples using the Tanaka formula. The general linear model (GLM) was applied to assess the association between SNPs and urinary sodium excretion or blood pressure. In the *SCNN1G* gene, six SNPs (rs4073291, rs12934362, rs7404408, rs4494543, rs5735, and rs6497657) were significantly different in 24-h urinary sodium excretion according to gene variants. However, no difference was found in blood pressure among participants with gene variants of ENaC. Our finding indicated that 24-h urinary sodium excretions were different according to variants of the *SCNN1G* gene in large samples. Further studies to replicate these findings are warranted.

## 1. Introduction

The epithelial sodium channel (ENaC) is expressed in several excretory organs such as the salivary glands, kidney, and lung [1,2,3,4,5,6]. There are four ENaC channel subunits, i.e., α, β, γ, and δ, in humans [7], which are encoded by four genes (*SCNN1A, SCNN1B, SCNN1G*, and *SCNN1D*, respectively).

ENaC is located in the distal nephron and plays a crucial role in controlling sodium balance. ENaC is mostly expressed in the luminal membrane of connecting tubule cells and plays an important role in renal sodium reabsorption and excretion [3,4]. Excessive sodium reabsorption by the kidney has been known to increase the risk of hypertension. In the kidney, the final control of sodium reabsorption takes place in the distal nephron through ENaC [4]. Liddle’s syndrome, a hereditary form of hypertension due to gain-of-function mutations in the genes coding for ENaC subunits, has shown the key role of ENaC in the sodium balance [8].

ENaC is located within taste cell membranes and is the primary mediator of salt taste. ENaC seems to be responsible for the appetitive behavioral responses caused by salt taste [1,2]. Taste perception plays an important role in determining individual food preferences, which may influence nutritional status and the risk of chronic disease [9]. If so, salt taste perception may affect sodium intake, which may further affect blood pressure.

ENaC has been reported to play an important role in lung fluid clearance [10]. *SCNN1A* appears to be essential in fluid transport in the lung. Genetic variants of *SCNN1A* were associated with the risk of neonatal respiratory distress syndrome [6].

Previous studies have suggested that genetic variants of ENaC subunits may influence blood pressure or hypertension [11]. The results are controversial. To our knowledge, the 24-h urinary sodium excretion by genetic variants of ENaC subunits has not been investigated yet among Asian populations. Therefore, this study was performed to determine whether genetic variation in ENaC (*SCNN1A*, *SCNN1B*, and *SCNN1G*) is associated with 24-h urinary sodium excretion and blood pressure in a Korean adult population.

## 2. Materials and Methods

### 2.1. Study Population

The KoGES_Ansan and Ansung study, an ongoing community-based cohort, was initiated to investigate the trends in diabetes and the related risk factors as part of the Korean Genome Epidemiology Study (KoGES) in 2001. At baseline, 10,038 participants aged 40–69 years were recruited from two communities, Ansan (*n* = 5020) and Ansung (*n* = 5018). Ansan is an urban area located in the southwest of Seoul (the capital of Korea), and Ansung is a rural area located south of Seoul. The baseline examination was conducted between 2001 and 2002, and the participants were followed up biennially. The details of the study design and procedures are described in a previous report [12].

DNA samples obtained from 10,004 participants were genotyped. After performing the sample and SNP quality control, 8840 participants were included in the data analysis. The detailed process has been described elsewhere [13]. Among 8840 participants, we excluded participants who had no information for spot urine samples (*n* = 4425). In addition, participants who were diagnosed by a doctor as having hypertension, diabetes, myocardial infarction, heart failure, coronary artery disease, stroke, renal disease, or cancer were excluded. Finally, a total of 3345 participants were included in the final data analysis. This study was approved by the electronic institutional review board (e-IRB) of the Korea National Institute for Bioethics Policy (KoNIBP).

### 2.2. General Characteristics and Anthropometric Variables

Interview-based questionnaires were performed by interviewers trained with a standardized manual to obtain demographic information, medical history, family history, and lifestyles. Weight was measured in kilograms to the nearest 0.1 kg, and height was measured within 0.1 cm without shoes. Body mass index (BMI) was defined as the body weight in kilograms divided by the square of the height in meters (kg/m^2^). Systolic blood pressure (SBP) and diastolic blood pressure (DBP) were measured at least twice in a supine position, and then an average value was calculated.

### 2.3. Collection of Urinary Samples and Estimation of 24-h Sodium Excretion

Urine specimens were self-collected in urine cups, and administrators transferred the urine into conical tubes, which were then sent to a central laboratory (Seoul Clinical Laboratories, Seoul, Korea) for measuring the quantities of sodium. Estimation of 24-h sodium excretion was performed using the Tanaka formula [14].

### 2.4. Genotyping

Genomic DNA samples were isolated from peripheral blood and genotyped on the Affymetrix Genome-Wide Human SNP Array 5.0 (Affymetrix Inc., Santa Clara, CA, USA). Genotype calling was conducted with the Bayesian Robust Linear Modeling using the Mahalanobis Distance (BRLMM) Genotyping Algorithm (DNA Link, Seoul, Korea) for 500,568 SNPs, and 352,228 SNPs remained after quality control. SNP imputation was conducted using the IMPUTE program [15,16]. Based on NCBI build 36 and dbSNP build 126, the JPT and CHB in HAPMAP were used as a reference panel comprising 3.99 million SNPs (HapMap release 22). All genetic variants were examined for Hardy–Weinberg equilibrium. Markers with Hardy–Weinberg equilibrium *p* value < 10^−6^ were discarded. More details on the genotype calling, quality-control, and imputation processes are described in a previous study [13].

### 2.5. Statistical Analysis

By using Plink (http://pngu.mgh.harvard.edu/~purcell/plink2/), we extracted SNP genotype data for gene regions (*SCNN1A*, *SCNN1B*, and *SCNN1G*) as additive (0, 1, and 2) components. A total of 34 SNPs were identified. Association analyses between SNPs and urinary sodium excretion or blood pressure were conducted using SAS software (version 9.3; SAS Institute, Cary, NC, USA).

The general linear model (GLM) was used to assess the association between SNPs and urinary sodium excretion or blood pressure according to the number of minor alleles after being adjusted for age, sex, BMI, and smoking status (non-smoker and current smoker). *p*-Values < 0.05 were considered significant. Tukey’s multiple comparison test was applied to search for specific differences between pairs of groups at *p* < 0.05. The linear trend test was performed by GLM in an additive genetic model with 1 degree of freedom.

## 3. Results

Table 1 shows the general characteristics of the study subjects. The mean ages of male and female subjects were 51.9 years and 51.0 years, respectively. The means of BMI for male and female subjects were 24.0 kg/m^2^ and 24.5 kg/m^2^. The proportions of the subjects with a family history of hypertension were 14.3% for male subjects and 15.6% for female subjects. Table 2 demonstrates allele distributions of SNPs in putative salt taste receptors within the study population. Major allele homozygous, heterozygous, and minor allele homozygous are presented as MM, Mm, and mm, respectively.

The associations of genetic variation in the ENaC gene with estimated 24-h urinary sodium excretion obtained with the Tanaka formula are presented in Table 3. In the *SCNN1G* gene, individuals homozygous for the C allele of rs4073291 (A>C) showed lower 24-h urinary sodium excretion than those with either the AA or AC genotype. Individuals with homozygous for the C allele of rs12934362 (T>C) showed lower 24-h urinary sodium excretion than those with either the TT or TC genotype. Individuals homozygous for the T allele of rs7404408 (C>T) showed lower 24-h urinary sodium excretion than those with either the CC or CT genotype. Individuals homozygous for the G allele of rs4494543 (A>G) showed lower 24-h urinary sodium excretion than those with either the AA or AG genotype. Individuals homozygous for the C allele of rs5735 (T>C) showed lower 24-h urinary sodium excretion than those with either the TT or TC genotype. Individuals homozygous for the C allele of rs6497657 (T>C) showed lower 24-h urinary sodium excretion than those with either the TT or TC genotype. In addition, as using the general linear model with an additive model after adjusting for age, sex, BMI, and smoking status, 24-h urinary sodium excretion showed significant decreasing trends in the *SCNN1G* gene (rs4073291, rs12934362, rs7404408, rs4494543, rs5735, rs6497657, rs4260062, rs5740, rs4470152, rs4309398, and rs4341748).

The associations of genetic variation in the ENaC gene with systolic blood pressure and diastolic blood pressure are shown in Table 4 and Table 5, respectively. No differences were found in systolic blood pressure and diastolic blood pressure among the genotype groups.

## 4. Discussion

The present study was performed to determine the association of genetic variation in ENaC (*SCNN1A*, *SCNN1B*, and *SCNN1G*) with urinary sodium excretion and blood pressure in Korean adults. Polymorphisms of six SNPs in the genes that code for the ENaC γ subunit (*SCNN1G*) may modify urinary sodium excretion. However, no difference was found in blood pressure among the gene variants of ENaC.

Homozygotes for the minor alleles of all SNPs in the *SCNN1G* gene showed lower 24-h urinary sodium excretion than carriers of major alleles. Among them, six SNPs (rs4073291, rs12934362, rs7404408, rs4494543, rs5735, and rs6497657) were significantly different in 24-h urinary sodium excretion and 11 SNPs (rs4073291, rs12934362, rs7404408, rs4494543, rs5735, rs6497657, rs4260062, rs5740, rs4470152, rs4309398, and rs4341748) showed significant decreasing trends in an additive model. However, there was no difference in the *SCNN1A* and *SCNN1B* genes.

ENaC is located in the distal nephron. Aldosterone and vasopressin regulate ENaC activity. A low-sodium diet stimulates the renin-angiotensin-aldosterone system (RAAS), and aldosterone increases sodium reabsorption by activating ENaC in the distal nephron. Vasopressin is secreted in response to increases in plasma osmolality, which means a water deficit. Vasopressin stimulates ENaC activity and increases water conservation by increasing sodium reabsorption [4]. In addition, proteolytic ENaC activation by serine proteases may contribute to sodium reabsorption [17,18,19]. Proteases cleave specific sites in the extracellular domains of the α- and γ-subunits [17,18,19].

Low 24-h urinary sodium excretion can be associated with high sodium reabsorption in the kidney or low sodium intake. Thus, the persons who are homozygous for the minor alleles of six SNPs (rs4073291, rs12934362, rs7404408, rs4494543, rs5735, and rs6497657) of the *SCNN1G* gene may reabsorb more sodium in the kidney or consume less sodium than the persons having carriers of major alleles. Excessive sodium reabsorption by the kidney has been known to increase the risk of hypertension. In a South African study, there were no differences in urinary sodium excretion between the wild type and carriers of the R563Q variant of the ENaC [20]. However, the G442V polymorphism in *SCNN1B* showed greater Na retention in normotensive young people (both black and white) [21]. In addition, Vormfelde SV et al. reported that carriers of the variant G-allele of rs5723 in *SCNN1G* tended to excrete less sodium in healthy white adults [22]. Therefore, variants of ENaC, particularly *SCNN1B* and *SCNN1G*, may be associated with reabsorption of more sodium, but there may be racial differences.

Twenty-four-hour urinary sodium excretion can reflect dietary sodium intake; thus, the people who are homozygous for the minor alleles of SNPs in the *SCNN1G* may consume less sodium than those who are carriers of major alleles. These differences seem to be related to taste perception. Salt taste perception has been the focus of studies on salt taste receptors such as the ENaC and transient receptor potential cation channel subfamily V member 1 (TRPV1) [23]. ENaC is located within taste cell membranes and is the key mediator of salt taste. In the study by Dias et al. [24], two SNPs (rs239345 (A>T) and rs3785368 (C>T)) in intronic regions within *SCNN1B* modified suprathreshold taste sensitivity. In the TRPV1 gene, one SNP (rs8065080 (C>T)) polymorphism modified salt taste perception. Carriers of the T allele perceived salt solutions stronger than those homozygous for the C allele. Even though the types of taste receptors were not the same as in the present study, genetic variation in salt taste receptors seems to modify taste perception, which results in the differences in salt intake. However, according to a twin study, salt taste perception appears to be determined more by environmental influences [25]. Further clinical trials on salt taste perception are needed.

In the present study, the *SCNN1G* gene was associated with 24-h urinary sodium excretion but was not related to blood pressure. The *SCNN1A* and *SCNN1B* genes were not associated with blood pressure either. The present study was performed to focus on urinary sodium excretion reflecting salt intake. Hypertensive patients were excluded from the study since they tried to modify their sodium intake. Previous studies reported conflicting results about the associations of ENaC with blood pressure [11,26,27,28,29]. The Genetic Epidemiology Network of Salt Sensitivity (GenSalt) study [26] conducted in the Han Chinese population reported that rs13306613 in the *SCNN1A* gene was associated with diastolic blood pressure (DBP) and that rs12447134 in the *SCNN1B* gene was associated with systolic blood pressure (SBP) under a codominant model. In addition, 5 SNPs in *SCNN1G* and 4 SNPs in *SCNN1B* were associated with SBP, DBP, or mean arterial pressure (MAP) under the additive model. In addition, in the other GenSalt study, *SCNN1A* SNP rs11064153 and *SCNN1G* SNP rs4401050 were related to longitudinal changes in SBP [11]. However, the T594M variant of the ENaC gene was not associated with hypertension among individuals of African ancestry [27] and Indo-Aryan ancestry [28]. The γ649 ENaC polymorphism was not related to hypertension or salt sensitivity either [29].

There are some limitations to consider when interpreting the results of this study. First, 24-h urinary sodium excretion was estimated using the Tanaka formula with spot urine samples. The 24-h urine collection is hardly realistic in large-sample epidemiologic studies. Second, sodium reabsorption in the kidney is affected by hormones, such as aldosterone and vasopressin, but they were not measured in the present study. Third, sodium intake and salt perception were not measured; thus, it is not known whether the difference in urinary sodium excretion according to ENaC gene variants is due to sodium intake (salt taste perception) or sodium reabsorption in the kidney. Fourth, persons with hypertension were excluded in the present study because hypertension patients tended to try a low sodium diet and we were more interested in sodium intake according to gene variants.

Despite these limitations, this study confirmed that there was a difference in 24-urinary sodium excretion according to variants of the *SCNN1G* gene in large samples, and this difference did not appear for blood pressure. Further studies are needed to determine whether the difference in 24-h urinary sodium excretion according to the ENaC gene variant is due to salt taste perception differences or sodium reabsorption differences in the kidney. In addition, since ENaC activity is also related to urinary potassium excretion, urinary Na/K ratio should be compared by genetic variation in the ENaC gene.

## Figures and Tables

**Table 1:** General characteristics of the study subjects.

Characteristics	Men	Women
*n*	1446	1899
Age (year)	51.9 ± 8.8 ^1^	51.0 ± 8.8
Income, ≥2,000,000 KRW (%)	33.3	30.0
Cigarette smoking, current (%)	55.7	3.6
Alcohol drinking, current (%)	68.9	25.3
Regular exercise, yes (%)	10.3	15.2
BMI (kg/m^2^) ^2^	24.0 ± 3	24.5 ± 3.2
Energy intake (kcal/day)	2115.5 ± 756.1	1980.2 ± 844.3
Family history of hypertension (%)	14.3	15.6
Blood pressure (mmHg)		
Systolic blood pressure	116.4 ± 15.8	113.9 ± 17.4
Diastolic blood pressure	75.4 ± 10.4	72.0 ± 11.2

^1^ Mean ± SD; ^2^ BMI: body mass index.

**Table 2:** The allele distributions of SNPs in the epithelial sodium channel (ENaC) within the study population.

Gene	SNP	Genotype ^1^			Alteration	MAF (%)
		MM	Mm	mm		
*SCNN1A*	rs13306613	3039 (90.85)	296 (8.85)	10 (0.30)	C>T	5
	rs7956915	1314 (39.29)	1564 (46.77)	466 (13.94)	G>A	37
	rs4149621	2823 (92.10)	238 (7.77)	4 (0.13)	T>C	4
	rs11064153	1391 (41.58)	1538 (45.98)	416 (12.44)	C>T	36
*SCNN1B*	rs7205273	1731 (51.75)	1334 (39.88)	280 (8.37)	C>T	27
	rs7190829	2152 (64.33)	1043 (31.18)	150 (4.48)	A>G	19
	rs8055868	2156 (64.49)	1030 (30.81)	157 (4.70)	G>A	19
	rs8044970	1064 (31.85)	1622 (48.55)	655 (19.6)	T>G	44
	rs152733	1100 (32.88)	1642 (49.09)	603 (18.03)	T>C	42
	rs239350	2682 (88.46)	336 (11.08)	14 (0.46)	C>T	6
	rs889299	1704 (50.97)	1346 (40.26)	293 (8.76)	G>A	30
	rs11074555	1921 (57.43)	1208 (36.11)	216 (6.46)	T>C	25
*SCNN1G*	rs4073291	2475 (73.99)	803 (24.01)	67 (2.00)	A>C	14
	rs12934362	2475 (74.84)	769 (23.25)	63 (1.91)	T>C	13
	rs7404408	2475 (74.21)	793 (23.78)	67 (2.01)	C>T	14
	rs4494543	2475 (74.21)	793 (23.78)	67 (2.01)	A>G	14
	rs5735	2462 (75.61)	734 (22.54)	60 (1.84)	T>C	13
	rs6497657	2457 (75.90)	721 (22.27)	59 (1.82)	T>C	13
	rs4299163	2796 (91.07)	267 (8.70)	7 (0.23)	G>C	5
	rs11643777	2962 (91.76)	261 (8.09)	5 (0.15)	C>G	4
	rs4260062	2797 (89.36)	325 (10.38)	8 (0.26)	T>C	6
	rs5740	2798 (89.08)	334 (10.63)	9 (0.29)	G>A	6
	rs4499238	2767 (84.36)	491 (14.97)	22 (0.67)	C>T	9
	rs4470152	2798 (87.63)	384 (12.03)	11 (0.34)	G>T	7
	rs4309398	2798 (87.79)	379 (11.89)	10 (0.31)	C>T	7
	rs4341748	2798 (87.82)	378 (11.86)	10 (0.31)	G>A	7
	rs11648257	2963 (89.22)	349 (10.51)	9 (0.27)	C>T	6
	rs9941210	2963 (89.19)	350 (10.54)	9 (0.27)	G>T	6
	rs4499239	2963 (89.17)	351 (10.56)	9 (0.27)	C>G	6
	rs13306653	2963 (88.90)	361 (10.83)	9 (0.27)	G>A	6
	rs11643517	2963 (88.77)	366 (10.96)	9 (0.27)	A>G	6
	rs5723	2961 (88.73)	367 (11.00)	9 (0.27)	C>G	6
	rs3026	2962 (88.74)	367 (10.99)	9 (0.27)	T>G	6
	rs9930846	2962 (88.74)	367 (10.99)	9 (0.27)	T>C	6

^1^ All values are presented as *n* (%); MM: major allele homozygous; Mm: heterozygous; mm: minor allele homozygous; MAF: minor allele frequency.

**Table 3:** Estimated 24-h urinary sodium excretion obtained with the Tanaka formula according to genotypes ^1^.

Gene	SNP	Genotype			*p*-Difference ^2^	*p*-Trend
		MM	Mm	mm		
*SCNN1A*	rs13306613	164.31 ± 0.66	163.10 ± 2.10	157.66 ± 11.25	0.7259	0.8506
	rs7956915	163.00 ± 1.00	164.43 ± 0.92	166.73 ± 1.66	0.1462	0.0926
	rs4149621	164.19 ± 0.69	162.84 ± 2.34	155.19 ± 17.84	0.7567	0.3700
	rs11064153	164.60 ± 0.97	163.49 ± 0.92	165.39 ± 1.78	0.5431	0.9487
*SCNN1B*	rs7205273	164.15 ± 0.87	164.14 ± 0.99	164.58 ± 2.15	0.9816	0.0521
	rs7190829	164.59 ± 0.78	163.54 ± 1.12	162.76 ± 2.94	0.6544	0.5149
	rs8055868	164.71 ± 0.78	163.05 ± 1.13	164.13 ± 2.88	0.4768	0.3728
	rs8044970	163.36 ± 1.11	164.59 ± 0.90	164.78 ± 1.41	0.6282	0.9313
	rs152733	164.27 ± 1.09	164.04 ± 0.89	164.41 ± 1.46	0.9722	0.4009
	rs239350	164.16 ± 0.70	164.51 ± 1.94	151.62 ± 9.81	0.4347	0.3109
	rs889299	164.12 ± 0.88	164.13 ± 0.98	165.02 ± 2.12	0.9214	0.3726
	rs11074555	164.20 ± 0.83	164.55 ± 1.04	161.94 ± 2.45	0.6170	0.0774
*SCNN1G*	rs4073291	164.43 ± 0.73 ^a 3^	164.37 ± 1.28 ^a^	152.25 ± 4.41 ^b^	**0.0241** ^4^	**0.0279**
	rs12934362	164.45 ± 0.73 ^a^	164.79 ± 1.31 ^a^	153.02 ± 4.57 ^b^	**0.0431**	**0.0331**
	rs7404408	164.44 ± 0.73 ^a^	164.53 ± 1.29 ^a^	152.26 ± 4.42 ^b^	**0.0237**	**0.0362**
	rs4494543	164.44 ± 0.73 ^a^	164.53 ± 1.29 ^a^	152.26 ± 4.42 ^b^	**0.0237**	**0.0362**
	rs5735	164.38 ± 0.73 ^a^	164.78 ± 1.34 ^a^	151.93 ± 4.69 ^b^	**0.0290**	**0.0209**
	rs6497657	164.36 ± 0.73 ^a^	164.62 ± 1.36 ^a^	151.06 ± 4.74 ^b^	**0.0198**	**0.0288**
	rs4299163	163.82 ± 0.69	163.91 ± 2.23	152.47 ± 13.46	0.7003	0.1345
	rs11643777	164.01 ± 0.67	166.33 ± 2.22	149.31 ± 17.79	0.4261	0.5863
	rs4260062	163.82 ± 0.69	163.44 ± 2.01	155.58 ± 12.57	0.7952	**0.0363**
	rs5740	163.83 ± 0.69	163.47 ± 1.98	159.05 ± 11.85	0.9096	**0.0432**
	rs4499238	163.93 ± 0.69	165.29 ± 1.63	160.32 ± 7.58	0.6561	0.2301
	rs4470152	163.83 ± 0.69	163.39 ± 1.85	160.11 ± 10.72	0.9202	**0.0497**
	rs4309398	163.83 ± 0.69	163.44 ± 1.86	158.62 ± 11.25	0.8827	**0.0351**
	rs4341748	163.83 ± 0.69	163.34 ± 1.86	158.61 ± 11.25	0.8735	**0.0319**
	rs11648257	163.97 ± 0.67	166.05 ± 1.92	151.15 ± 12.57	0.3458	0.7657
	rs9941210	163.96 ± 0.67	166.04 ± 1.92	151.15 ± 12.56	0.3467	0.7642
	rs4499239	163.97 ± 0.67	165.98 ± 1.92	151.15 ± 12.56	0.3567	0.7705
	rs13306653	163.97 ± 0.67	166.56 ± 1.89	151.16 ± 12.58	0.2524	0.9566
	rs11643517	163.96 ± 0.67	166.31 ± 1.88	151.16 ± 12.58	0.2919	0.9887
	rs5723	163.95 ± 0.67	166.43 ± 1.87	151.17 ± 12.57	0.2681	0.9409
	rs3026	163.95 ± 0.67	166.43 ± 1.87	151.17 ± 12.57	0.2668	0.9361
	rs9930846	163.95 ± 0.67	166.43 ± 1.87	151.17 ± 12.57	0.2668	0.9361

^1^ All values are presented as the mean ± SD (mEq/day); ^2^ Values were derived using a general linear model analysis adjusted for age, sex, BMI, and smoking status; ^3^ Values with different superscript letters within a row are significantly different (*p* < 0.05) among groups by Tukey’s multiple comparison test; ^4^ The significant *p* value was denoted in bold; MM: major allele homozygous; Mm: heterozygous; mm: minor allele homozygous.

**Table 4:** Associations between systolic blood pressure and genotypes ^1^.

Gene	SNP	Genotype			*p*-Difference ^2^	*p*-Trend
		MM	Mm	mm		
*SCNN1A*	rs13306613	114.99 ± 0.28	114.36 ± 0.89	114.38 ± 4.80	0.7928	0.5030
	rs7956915	115.11 ± 0.43	114.57 ± 0.39	115.66 ± 0.71	0.3507	0.8536
	rs4149621	114.86 ± 0.29	113.99 ± 0.99	108.12 ± 7.57	0.4773	0.3007
	rs11064153	114.71 ± 0.41	115.09 ± 0.39	115.09 ± 0.76	0.7767	0.5310
*SCNN1B*	rs7205273	115.16 ± 0.37	114.84 ± 0.42	113.96 ± 0.92	0.4596	0.2428
	rs7190829	115.16 ± 0.33	114.43 ± 0.48	115.12 ± 1.26	0.4372	0.3529
	rs8055868	115.14 ± 0.33	114.29 ± 0.48	116.31 ± 1.23	0.1789	0.6593
	rs8044970	115.25 ± 0.47	114.85 ± 0.38	114.59 ± 0.60	0.6646	0.3710
	rs152733	114.69 ± 0.47	115.02 ± 0.38	115.11 ± 0.62	0.8160	0.5502
	rs239350	114.86 ± 0.30	115.45 ± 0.83	112.29 ± 4.22	0.6594	0.6956
	rs889299	115.05 ± 0.38	114.83 ± 0.42	114.63 ± 0.91	0.8739	0.6036
	rs11074555	114.81 ± 0.35	115.39 ± 0.44	113.48 ± 1.05	0.2107	0.8865
*SCNN1G*	rs4073291	114.75 ± 0.31	115.43 ± 0.55	115.61 ± 1.88	0.5250	0.2658
	rs12934362	114.80 ± 0.31	115.46 ± 0.56	116.20 ± 1.94	0.4706	0.2196
	rs7404408	114.77 ± 0.31	115.45 ± 0.55	115.62 ± 1.88	0.5213	0.2637
	rs4494543	114.77 ± 0.31	115.45 ± 0.55	115.62 ± 1.88	0.5213	0.2637
	rs5735	114.78 ± 0.31	115.39 ± 0.57	116.48 ± 1.99	0.4694	0.2250
	rs6497657	114.78 ± 0.31	115.22 ± 0.58	116.12 ± 2.01	0.6577	0.3715
	rs4299163	114.97 ± 0.29	114.78 ± 0.95	112.20 ± 5.73	0.8755	0.7415
	rs11643777	114.94 ± 0.29	115.30 ± 0.95	110.52 ± 7.60	0.7898	0.8350
	rs4260062	114.95 ± 0.29	114.17 ± 0.86	112.04 ± 5.35	0.6009	0.3270
	rs5740	114.95 ± 0.29	114.35 ± 0.85	114.15 ± 5.05	0.7879	0.4925
	rs4499238	115.01 ± 0.30	114.81 ± 0.70	111.91 ± 3.25	0.6187	0.5358
	rs4470152	114.93 ± 0.29	114.20 ± 0.79	114.37 ± 4.56	0.6794	0.3903
	rs4309398	114.93 ± 0.29	114.25 ± 0.79	113.17 ± 4.79	0.6764	0.3784
	rs4341748	114.93 ± 0.29	114.30 ± 0.79	113.18 ± 4.79	0.7057	0.4068
	rs11648257	114.92 ± 0.29	115.10 ± 0.82	109.26 ± 5.37	0.5605	0.9094
	rs9941210	114.92 ± 0.29	115.09 ± 0.82	109.26 ± 5.37	0.5622	0.8986
	rs4499239	114.92 ± 0.29	115.05 ± 0.82	109.26 ± 5.37	0.5668	0.8635
	rs13306653	114.92 ± 0.29	115.08 ± 0.81	109.25 ± 5.37	0.5615	0.8993
	rs11643517	114.90 ± 0.29	115.03 ± 0.80	109.23 ± 5.36	0.5638	0.8768
	rs5723	114.90 ± 0.29	115.12 ± 0.80	109.23 ± 5.36	0.5502	0.9617
	rs3026	114.91 ± 0.29	115.13 ± 0.80	109.24 ± 5.36	0.5514	0.9547
	rs9930846	114.91 ± 0.29	115.13 ± 0.80	109.24 ± 5.36	0.5514	0.9547

^1^ All values are presented as the mean ± SD (mmHg); ^2^ Values were derived using a general linear model analysis adjusted for age, sex, BMI, and smoking status; MM: major allele homozygous; Mm: heterozygous; mm: minor allele homozygous.

**Table 5:** Associations between diastolic blood pressure and genotypes ^1^.

Gene	SNP	Genotype			*p* -Difference ^2^	*p* -Trend
		MM	Mm	mm		
*SCNN1A*	rs13306613	73.76 ± 0.19	72.43 ± 0.61	75.20 ± 3.26	0.1004	0.0728
	rs7956915	73.57 ± 0.29	73.54 ± 0.27	74.22 ± 0.48	0.4435	0.3752
	rs4149621	73.63 ± 0.20	73.05 ± 0.67	69.28 ± 5.15	0.4974	0.3078
	rs11064153	73.49 ± 0.28	73.66 ± 0.27	74.13 ± 0.51	0.5535	0.3084
*SCNN1B*	rs7205273	73.80 ± 0.25	73.56 ± 0.29	73.12 ± 0.62	0.5585	0.2917
	rs7190829	73.75 ± 0.23	73.39 ± 0.32	73.99 ± 0.85	0.6028	0.6225
	rs8055868	73.70 ± 0.23	73.40 ± 0.33	74.75 ± 0.83	0.3014	0.8278
	rs8044970	73.63 ± 0.32	73.70 ± 0.26	73.49 ± 0.41	0.9067	0.8237
	rs152733	73.40 ± 0.32	73.87 ± 0.26	73.49 ± 0.42	0.4675	0.6841
	rs239350	73.61 ± 0.20	73.88 ± 0.56	72.55 ± 2.85	0.8360	0.7733
	rs889299	73.75 ± 0.25	73.53 ± 0.28	73.54 ± 0.62	0.8214	0.5754
	rs11074555	73.74 ± 0.24	73.67 ± 0.30	72.72 ± 0.71	0.3968	0.3177
*SCNN1G*	rs4073291	73.63 ± 0.21	73.61 ± 0.37	74.66 ± 1.28	0.7254	0.7256
	rs12934362	73.65 ± 0.21	73.78 ± 0.38	75.16 ± 1.31	0.5145	0.4168
	rs7404408	73.64 ± 0.21	73.64 ± 0.37	74.67 ± 1.28	0.7285	0.6950
	rs4494543	73.64 ± 0.21	73.64 ± 0.37	74.67 ± 1.28	0.7285	0.6950
	rs5735	73.65 ± 0.21	73.76 ± 0.39	74.99 ± 1.35	0.6094	0.4860
	rs6497657	73.64 ± 0.21	73.77 ± 0.39	74.74 ± 1.36	0.7020	0.5164
	rs4299163	73.72 ± 0.20	73.47 ± 0.65	72.98 ± 3.92	0.9172	0.6805
	rs11643777	73.72 ± 0.19	73.62 ± 0.64	70.41 ± 5.14	0.8056	0.7673
	rs4260062	73.71 ± 0.20	73.15 ± 0.59	72.60 ± 3.66	0.6350	0.3405
	rs5740	73.71 ± 0.20	73.25 ± 0.58	72.55 ± 3.46	0.7146	0.4139
	rs4499238	73.73 ± 0.20	73.27 ± 0.47	71.72 ± 2.20	0.4578	0.2441
	rs4470152	73.70 ± 0.20	73.18 ± 0.54	72.76 ± 3.12	0.6310	0.3374
	rs4309398	73.71 ± 0.20	73.17 ± 0.54	71.94 ± 3.27	0.5620	0.2915
	rs4341748	73.71 ± 0.20	73.18 ± 0.54	71.94 ± 3.27	0.5771	0.3041
	rs11648257	73.71 ± 0.19	73.20 ± 0.56	69.92 ± 3.65	0.4045	0.2567
	rs9941210	73.71 ± 0.19	73.20 ± 0.56	69.92 ± 3.65	0.4060	0.2586
	rs4499239	73.72 ± 0.19	73.21 ± 0.56	69.92 ± 3.65	0.4099	0.2633
	rs13306653	73.71 ± 0.19	73.20 ± 0.55	69.92 ± 3.65	0.4018	0.2555
	rs11643517	73.71 ± 0.19	73.19 ± 0.54	69.90 ± 3.64	0.3956	0.2494
	rs5723	73.7 ± 0.19	73.19 ± 0.54	69.90 ± 3.64	0.3966	0.2503
	rs3026	73.71 ± 0.19	73.19 ± 0.54	69.91 ± 3.64	0.3941	0.2477
	rs9930846	73.71 ± 0.19	73.19 ± 0.54	69.91 ± 3.64	0.3941	0.2477

^1^ All values are presented as the mean ± SD (mmHg); ^2^ Values were derived using a general linear model analysis adjusted for age, sex, BMI, and smoking status; MM: major allele homozygous; Mm: heterozygous; mm: minor allele homozygous.

## References

[1] DeSimone J.A., Lyall V. (2006). Taste receptors in the gastrointestinal tract III. Salty and sour taste: Sensing of sodium and protons by the tongue. Am. J. Physiol. Gastrointest. Liver Physiol..

[2] Chandrashekar J., Kuhn C., Oka Y., Yarmolinsky D.A., Hummler E., Ryba N.J., Zuker C.S. (2010). The cells and peripheral representation of sodium taste in mice. Nature.

[3] Thomas C.P., Itani O.A. (2004). New insights into epithelial sodium channel function in the kidney: Site of action, regulation by ubiquitin ligases, serum- and glucocorticoid-inducible kinase and proteolysis. Curr. Opin. Nephrol. Hypertens..

[4] Bankir L., Bichet D.G., Bouby N. (2010). Vasopressin V2 receptors, ENaC, and sodium reabsorption: A risk factor for hypertension?. Am. J. Physiol. Ren. Physiol..

[5] Huppert L.A., Matthay M.A. (2017). Alveolar fluid clearance in pathologically relevant conditions: In vitro and in vivo models of acute respiratory distress syndrome. Front. Immunol..

[6] Li W., Long C., Renjun L., Zhangxue H., Yin H., Wanwei L., Juan M., Yuan S. (2015). Association of SCNN1A Single Nucleotide Polymorphisms with neonatal respiratory distress syndrome. Sci. Rep..

[7] Kellenberger S., Schild L. (2002). Epithelial sodium channel/degenerin family of ion channels: A variety of functions for a shared structure. Physiol. Rev..

[8] Shimkets R.A., Warnock D.G., Bositis C.M., Nelson-Williams C., Hansson J.H., Schambelan M., Gill J.R., Ulick S., Milora R.V., Findling J.W. (1994). Liddle’s syndrome: Heritable human hypertension caused by mutations in the beta subunit of the epithelial sodium channel. Cell.

[9] Garcia-Bailo B., Toguri C., Eny K.M., El-Sohemy A. (2009). Genetic variation in taste and its influence on food selection. Omics J. Integr. Biol..

[10] Folkesson H.G., Matthay M.A. (2006). Alveolar epithelial ion and fluid transport: Recent progress. Am. J. Respir. Cell Mol. Biol..

[11] Yang X., He J., Gu D., Hixson J.E., Huang J., Rao D.C., Shimmin L.C., Chen J., Rice T.K., Li J. (2014). Associations of epithelial sodium channel genes with blood pressure changes and hypertension incidence: The GenSalt study. Am. J. Hypertens..

[12] Kim Y., Han B.G. (2017). Cohort profile: The Korean Genome and Epidemiology Study (KoGES) Consortium. Int. J. Epidemiol..

[13] Cho Y.S., Go M.J., Kim Y.J., Heo J.Y., Oh J.H., Ban H.J., Yoon D., Lee M.H., Kim D.J., Park M. (2009). A large-scale genome-wide association study of Asian populations uncovers genetic factors influencing eight quantitative traits. Nat. Genet..

[14] Tanaka T., Okamura T., Miura K., Kadowaki T., Ueshima H., Nakagawa H., Hashimoto T. (2002). A simple method to estimate populational 24-h urinary sodium and potassium excretion using a casual urine specimen. J. Hum. Hypertens..

[15] Marchini J., Howie B., Myers S., McVean G., Donnelly P. (2007). A new multipoint method for genome-wide association studies by imputation of genotypes. Nat. Genet..

[16] Wellcome Trust Case Control Consortium (2007). Genome-wide association study of 14,000 cases of seven common diseases and 3000 shared controls. Nature.

[17] Bruns J.B., Carattino M.D., Sheng S., Maarouf A.B., Weisz O.A., Pilewski J.M., Hughey R.P., Kleyman T.R. (2007). Epithelial Na+ channels are fully activated by furin- and prostasin-dependent release of an inhibitory peptide from the gamma-subunit. J. Biol. Chem..

[18] Carattino M.D., Sheng S., Bruns J.B., Pilewski J.M., Hughey R.P., Kleyman T.R. (2006). The epithelial Na+ channel is inhibited by a peptide derived from proteolytic processing of its alpha subunit. J. Biol. Chem..

[19] Kleyman T.R., Carattino M.D., Hughey R.P. (2009). ENaC at the cutting edge: Regulation of epithelial sodium channels by proteases. J. Biol. Chem..

[20] Jones E.S., Owen E.P., Rayner B.L. (2012). The association of the R563Q genotype of the ENaC with phenotypic variation in Southern Africa. Am. J. Hypertens..

[21] Ambrosius W.T., Bloem L.J., Zhou L., Rebhun J.F., Snyder P.M., Wagner M.A., Guo C., Pratt J.H. (1999). Genetic variants in the epithelial sodium channel in relation to aldosterone and potassium excretion and risk for hypertension. Hypertension.

[22] Vormfelde S.V., Sehrt D., Toliat M.R., Schirmer M., Meineke I., Tzvetkov M., Nurnberg P., Brockmoller J. (2007). Genetic variation in the renal sodium transporters NKCC2, NCC, and ENaC in relation to the effects of loop diuretic drugs. Clin. Pharmacol. Ther..

[23] Chamoun E., Mutch D.M., Allen-Vercoe E., Buchholz A.C., Duncan A.M., Spriet L.L., Haines J., Ma D.W.L. (2018). A review of the associations between single nucleotide polymorphisms in taste receptors, eating behaviors, and health. Crit. Rev. Food Sci. Nutr..

[24] Dias A.G., Rousseau D., Duizer L., Cockburn M., Chiu W., Nielsen D., El-Sohemy A. (2013). Genetic variation in putative salt taste receptors and salt taste perception in humans. Chem. Senses.

[25] Wise P.M., Hansen J.L., Reed D.R., Breslin P.A. (2007). Twin study of the heritability of recognition thresholds for sour and salty taste. Chem. Senses.

[26] Liu F., Yang X., Mo X., Huang J., Chen J., Kelly T.N., Hixson J.E., Rao D.C., Gu C.C., Shimmin L.C. (2015). Associations of epithelial sodium channel genes with blood pressure: The GenSalt study. J. Hum. Hypertens..

[27] Nkeh B., Samani N.J., Badenhorst D., Libhaber E., Sareli P., Norton G.R., Woodiwiss A.J. (2003). T594M variant of the epithelial sodium channel beta-subunit gene and hypertension in individuals of African ancestry in South Africa. Am. J. Hypertens..

[28] Gupta M.D., Girish M.P., Sikdar S., Ahuja R., Shah D., Kumar R., Rain M., Nejatizadeh A., Tyagi S., Pasha Q. (2014). beta-T594M epithelial sodium channel gene polymorphism and essential hypertension in individuals of Indo-Aryan ancestry in Northern India. Indian Heart J..

[29] Poch E., Gonzalez D., de la Sierra A., Giner V., Bragulat E., Botey A., Coca A., Rivera F. (2000). Genetic variation of the gamma subunit of the epithelial Na+ channel and essential hypertension. Relationship with salt sensitivity. Am. J. Hypertens..

